# Use of tianeptine sulfate over-the-counter, literature review, and case report

**DOI:** 10.1192/j.eurpsy.2023.347

**Published:** 2023-07-19

**Authors:** A. J. Palma Conesa, M. I. Rico Rangel, I. González Milla, A. Aranzadi

**Affiliations:** Psychiatry, Servicio Andaluz de Salud. Área de Gestión Sanitaria de Osuna, Sevilla, Spain

## Abstract

**Introduction:**

Tianeptine is prescribed as an antidepresant in Europe. The prescription drug is produced as tianeptine sodium. However, it can also be found on the Internet as tianeptine sulfate sold as a nootropric. Misuse of tianeptine sodium has been documented, but there’s little scientific evidence of tianeptine sulfate use.

**Objectives:**

Review the use of tianeptine sulfate without prescription.

Present a clinical report of tianeptine sulfate use.

**Methods:**

PubMed review of tienptine use without prescription.

Clinical report of a patient using tianeptine sulfate adquiered on the Internet.

**Results:**

Systematic review on PubMed using the search term “tianeptine abuse” conducted on 01/10/2022. A total of 71 articles were found from wich 33 mentioned the use of tianeptine use without prescription. A total of 23 case reports of tianeptine use without prescription were found. None of them made the difference between tiaenptine sodium or tianeptine sulfate. Only one article mentioned the use of tianeptine sulfate from an Internet search on Internet fora (Smith et al. Am J Drug Alcohol Abuse 2021 47(4), 455–466).

The case report of a 23 years old patient is presented. Diagnoses: ADHD (F98.8); Psychotic episodes four (F23) and two (F16.150) years ago; Major depressive disorder (F32.2). Use of psychedelics, cannabis, psychoestimulants and opioids meeting substance use disorder (SUD) criteria. The patient brought proof of the tianeptine sulfate bought on the Internet (image 1) for self-treatment of his depressive symptoms. Tianeptine sodium is prescribed. The patient then restarts opioid use with fear of a new opioid use disorder episode and an oxicodone prescription is maintained. The patient then interrupts the antipsychotic medication and suffers a psychotic relapse. After this psychotic episode a LAI treatment with paliperidone is started. Currently, the patient is recovered and maintains psychopathological stability and abstinence from other substances. Blod test result unaltered. Current treatment: lisdexamphetamine 70mg /day, tianeptina sodium 12.5mg/day; oxicodone 40mg/12h and paliperidone 150mg /28d.

**Image:**

**Figure:**
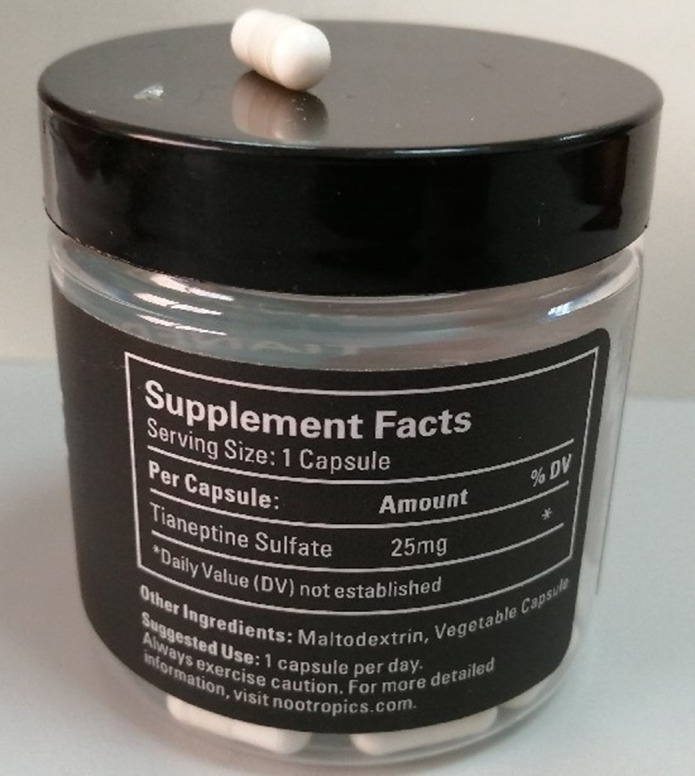

**Conclusions:**

Tianeptine sulfate is believed to present some diferences versus tianeptine sodium: it is sold only through the Internet without prescrption, the daily dose is 25mg per day versus 12.5mg every 8 hours and it might be more potent and long lasting. Those differences were confirmed by the patient after the prescription of tianeptine sodium.

Self-medication with psychoactive substances is one of the theories for substance use and might lead to a substance use disorder. This case shows how the prescription of a drug similar to the one used without presription might favour the therapeutical alliance and reduce the risk associated to the use of non-regulated substances.

Further research is needed to better understand the use of tianeptine sulfate.

**Disclosure of Interest:**

None Declared

